# Identification of Active Compounds From Yi Nationality Herbal Formula *Wosi* Influencing COX-2 and VCAM-1 Signaling

**DOI:** 10.3389/fphar.2020.568585

**Published:** 2020-11-09

**Authors:** Ji-Zhong Zhang, Xiao-Yi Chen, You-Jiao Wu, Li-Min Li, Li Huang, Qiao-Zhi Yin, Pei Luo, Yuan Liu

**Affiliations:** ^1^ Institute of Ethnic Medicine, Southwest Minzu University, Chengdu, China; ^2^ Sichuan Provincial Qiang-Yi Medicinal Resources Protection and Utilization Technology and Engineering Laboratory, Chengdu, China; ^3^ State Key Laboratories for Quality Research in Chinese Medicines, Macau University of Science and Technology, Macau, China; ^4^ Sichuan Academy of Chinese Medicine Sciences, Chengdu, China; ^5^ Chengdu University of TCM, Chengdu, China

**Keywords:** yi nationality herbal formula *wosi*, acute gouty arthritis, hyperuricemia, COX-2, VCAM-1

## Abstract

The Yi nationality herbal formula *Wosi* is used in China as a folk medicine to treat arthritis and related diseases. Despite its widespread use, the active ingredients, and pharmacological mechanisms are not performed. This is the first time to identify the active compounds from *Wosi* with the aim at providing the potential effect of *Wosi* and exploring its underlying anti-inflammatory mechanism in monosodium urate crystals (MSU)-induced arthritis rats. In this study, anti-hyperuricemia effect was assessed by reducing the serum uric acid levels and increasing uric acid excretion in the urine for the hyperuricemia rat model. *Wosi* significantly suppressed the degree of joint swelling and improved the symptoms of inflammation induced by MSU crystals. The inhibition of IL-2, IL-1β, IFN-γ, and IL-6 secretion and IL-10 increase in the serum were also observed. This study also focuses on the screening of the main compounds from *Wosi* against cyclooxygenase for anti-inflammatory properties using molecular docking. The result showed 3-O-[α-L-pyran rhamnose(1-3)-β-D-pyran glucuronic acid]- oleanolic acid, 3-O-(β-D-pyran glucuronic acid)-oleanolic acid-28-O-β-D-pyran glucoside, and 3-O-[α-L-pyran rhamnose(1-3)-β-D-pyran glucuronic acid]-oleanolic acid-28-O-β-D-pyran glucoside with a higher binding affinity for COX-2 than COX-1 which indicated relatively higher interaction than COX-1. The preferential selectivity toward inhibiting COX-2 enzyme over COX-1 of three compounds from *Wosi* were evaluated using *in-vitro* cyclooxygenases 1 and 2 (COX-1/2) inhibition assays. Meanwhile, the down-regulated protein expression of COX-2 and VCAM-1 in synovial tissue sections from ankle joints of experiments rats were confirmed by immunohistochemistry analysis after the *Wosi* treatment. In conclusion, three oleanolic acid glycosides were implied as mainly efficient compounds in Yi nationality herbal formula *Wosi* for arthritis therapy via selectively influencing COX-2 and VCAM-1 signaling.

## Introduction

Hyperuricemia associated with the overproduction and insufficient excretion of uric acid. The incidence of hyperuricemia in the world is gradually increasing, which is a risk factor for development of gout, cardiovascular disease, and diabetes (Falasca, 2006; Johnson et al., 2013). Moreover, high concentration of uric acid in the blood may result in monosodium urate crystals (MSU) deposition in the joint tissues and synovial membrane causing acute gout arthritis. It is mainly manifested in redness, swelling, heat, and pain in the joints due to various inflammatory mediators. The recognition of MSU crystals can trigger innate host defense responses through Toll-like receptor (TLR)_2_ and (TLR)_4_. TLR_2_, TLR_4_ and the TLR adaptor protein MyD88 can promote the phagocytosis of MSU crystals surrounding in the joints by phagocytes (Martinon et al., 2006; Sabina et al., 2011; Rock et al., 2013). When macrophage engulfs MSU crystals, it promotes the assembly and the activation of cytosolic NACHT-LRR-PYD-containing protein (NLRP3) inflammasome protein complex (Cronstein and Terkeltaub, 2006; So and Martinon, 2017). Inflammasome in response to MSU crystals trigger caspase-1 activation and the maturation and release of interleukin 1 beta (IL-1β) in phagocytes, it also can promote the inflammation response by the release of cytokines,chemokines and expression of inflammatory enzymes (cyclooxygenase-2) and inducible nitric oxide synthase (iNOS) (Kinne et al., 2000; Pétrilli and Martinon, 2007). This process could cause monocytes and T lymphocytes in blood flowing into the site of inflammation and enhance inflammatory reactions by activating the expression of Vascular cell adhesion protein 1 (VCAM-1) (Carlos et al., 1991). Classically, the major therapeutic approaches against gouty arthritis is to control serum urate concentration in a controllable level and treat the inflammation. At present, clinical treatment of gouty arthritis mainly based on non-steroidal anti-inflammatory drugs (indomethacin and celecoxib) as well as urate-lowering (benzbromarone and allopurinol) as the first line therapies (Holmdahl et al., 2006; Neogi, 2011; Gliozzi et al., 2016). In general, non-steroidal anti-inflammatory drugs (NSAIDs) exert its pharmacological activity by inhibiting cyclooxygenase (COX), which has at least two main isoforms, cyclooxygenase-1 (COX-1) and cyclooxygenase-2 (COX-2). The expression of these two isoforms is differentially regulated. Their anti-inflammatory effects are mainly due to inhibition of COX-2 (Fam, 2002). COX-1 is a constitutive enzyme that can maintain the vascular homeostasis but COX-2 can only be induced to express after inflammatory stimulation (Zidar et al., 2009). However, the long-term used of NSAIDs may cause gastrointestinal discomfort due to the inhibition of COX-1 (Adib-Conquy et al., 1999; van Echteld et al., 2014), which limit their clinical application. Therefore, it is still meaningful to find some alternative drugs which have better selectivity for COX-2 enzyme to intervene in acute gouty arthritis and hyperuricemia.

The development of Yi medicine has spanned thousands of years from primitive society to modern times which is an integral part of Chinese ethnic medicine. It is still widely used nowadays for Yi people to against diseases. *Wosi* have consist of five herbs including *Cyathula capitata* (Wall.) Moq., *Sargentodoxa cuneata* (Oliv.) Rehd. et Wils., *Dipsacus asperoides* C. Y. Cheng et T., *GaultherialeucocarpaBl*. var. *crenulata* T. Z. Hsu, and *Dioscorea collettii* Hook. f. var. *hypoglauca (Palibin)* C. T. Ting et al. *Wosi* is one of the most applied formulations which is believed to have therapeutic effects on arthritis and its related diseases. The clinical applications have demonstrated the therapeutic effects. However, as far as we know, there is no scientific data exists to support the therapeutic effects and active ingredients of *Wosi*. Therefore, this study test whether *Wosi* have underlying anti-inflammatory and anti-hyperuricemia properties to ameliorate hyperuricemia and acute gouty arthritis and to imply the efficient compounds from *Wosi* for the first time.

## Materials and Methods

### Chemical and Reagents

*Wosi* was provided by the Institute of Yi Medicine at Liangshan (Sichuan, China). Colchicine tablets were purchased from Dianhong Pharmaceutical Co., Ltd. (Yunnan, China). Benzbromarone tablets were purchased from Yichang Hec Changjiang Pharmaceutical Co., Ltd. (Sichuan, China). Adenine (purity ≥ 99%) was purchased from Sangon biotech Co., Ltd. (Shanghai, China). Rabbit IgG Immunohistochemical kit; 3,3′-diaminobenzidine (DAB) kit; antibodies against VCAM-1 and COX-2, and enzyme-linked immunosorbent assay (ELISA) kits for IL-6, IL-1β, IL-2, IFN-γ, and IL-10 were purchased from Wuhan Boster Bio-engineering Co., Ltd. (Hubei, China). Uric acid assay kits were purchased from Nanjing Jiancheng Bio-engineering Co., Ltd. (Jiangsu, China). Uric acid sodium salt was purchased from Sigma (St. Louis, MO, USA). All other reagents used were standard laboratory reagents of analytical grade and were purchased locally.

### Animals

Male Sprague-Dawley (SD) rats (200–250 g) were supplied by the Animal Center of Sichuan Academy of Chinese Medicine Sciences, Chengdu. Animals were housed in plastic cages and raised at 18–22 C and the relative humidity was set as 40–70%. Animals maintained on a 12/12 h light/dark cycle. In the duration of the study, it was fed with standard food and free access water. All the animal experiments were performed in compliance with the institutional ethics committee regulations and guidelines on animal welfare (Animal Care and Use Program Guidelines of Sichuan Academy of Chinese Medicine Sciences).

### Preparation of Drugs

Five samples S1-S5 consisted of *Wosi* were collected from Xicang, Sichuan Province, China. The plant materials were authenticated by Prof. Hao Zhang (Professor of pharmacognosy, School of Pharmacy, Sichuan University, Chengdu, China). The voucher specimens (Supplementary Table S1) were deposited in the Herbarium Center, Southwest University for Nationalities, Sichuan, China. *Wosi* powder was boiled with water twice for 30 min, combined with decoction at 60°C, and concentrated to the volume that equaled to 1 g powder per ml as the storage formulation. Colchicine was prepared in suspension with 0.9% sterile saline. Benzbromarone was prepared in suspension with 0.5% carboxymethyl cellulose sodium salt (CMC-Na). MSU was suspended in 0.9% sterile saline (25 mg/ml) immediately before its used.

### Model of Hyperuricemia and Drug Treatment

The rats were divided into six groups, and each group comprised of 10 animals which were control, model, Benzbromarone (0.01 mg/kg), *Wosi* (0.5, 1, and 2 g/kg) groups. Briefly, 10% dry yeast solution of 1.25% adenine (10 ml/kg) was given orally administration for 15 consecutive days to induce the hyperuricemia in each animal except those of control groups. The solution of 0.5% CMC-Na was given to the control groups. On the 10th day, the *Wosi* and Benzbromarone were orally administered continuously 24 h 5 days. Normal saline was given to the control and model groups. Collecting 14th and 15th days of rat urine (24 h) and recording the volume. It was centrifuged at 3,500 g for 5 min to remove the precipitations. On the 15th day, rats were first anesthetized with pentobarbital sodium (i.p., 70 mg/kg body weight), 1 h after the final drug treatment. Then the blood sample was collected and centrifuged at 3000 g for 5 min to obtain the serum. Serum were kept under −20°C.

### Uric Acid Assay

According to manufacturer’s instructions, using a standard diagnostic kit, was used in order to evaluate the concentration of uric acid concentration in serum and urine.

### Model of Acute Gouty Arthritis and Drug Treatment

Rats were stochastically divided into six groups and each group comprised of 10 animals which were control; model; colchicine (0.5 mg/kg), *Wosi* (0.5, 1, and 2 g/kg) groups. *Wosi* was orally administration once daily for seven consecutive days in *Wosi* group. Meanwhile, colchicine was orally administration once daily for seven consecutive days in colchicine groups. The solution of 0.5% CMC-Na was given to the control and model groups. MSU was injected for 0.05 ml (25 mg/ml) in the left ankle joint except those of control groups to induced acute gouty arthritis in the rat, 1 h after the last orally administration of *Wosi* and colchicine on the seventh day. 0.9% sterile saline (0.05 ml) was given to the control group.

### Assessment of Joint Swelling

After injection of MSU crystal, the joint swelling of arthritis was assessed by measuring the diameter of the left ankle joint with inelastic soft ruler at 0, 1, 3, 4, 5, 6, 7, 8, and 24 h. The change in joint swelling was calculated as follows: joint swelling = *b*—a. Here, a is the initial diameter of rat knee joint; b is the diameter after the injection of MSU crystal at each time point.

### Blood and Tissue Processing

Rats were divided as aforementioned. *Wosi* at three doses (0.5, 1, and 2 g/kg) were orally administration once daily for 7 days in *Wosi* groups. Meanwhile, colchicine was orally administration once daily for 7 days in colchicine groups. The injection of the MSU crystal was performed on the fifth day for each animal except those of control groups. 0.9% sterile saline was given to the control group. On the seventh day, rats were first anesthetized with pentobarbital sodium (i.p., 70 mg/kg body weight) at 30 min after the last dosing, Blood samples were taken from rat femoral artery with heparinized needles which were allowed to clot for 1 h at room temperature and centrifuged at 3,000 g for 10 min to obtain serum. Serum samples were stored at −20°C until the ELISA testing, then rats were sacrificed by femoral artery exsanguination. Meanwhile, the ankle joint surrounding tissues were collected for the immunohistochemical analyze and frozen at −80°C.

### Cytokines Assay

The levels of interleukin 6 (IL-6), interleukin 1 beta, interleukin 2 (IL-2), interleukin 10 (IL-10), and interferon gamma (IFN-γ) in the serum were measured using ELISA kits according to manufacturer’s instructions.

### Extraction and Isolation of Active Compounds

The root of *Cyathula capitata* (Wall.) Moq. (1,000 g) which is one of the main herbs in Yi nationality herbal formula *Wosi* were finely powdered in a grinder (24 mesh). Next, 80% ethanol was added according to the liquid-to-solid ratio of 1:20 (ml g^−1^), extracted at 90°C for 60 min each time (3 times), and filtered while hot. The filtrate was decompressed and concentrated to the ethanol volatilized completely (80% ethanol extract), then dissolved by 500 ml distilled water. The aqueous phase was extracted with petroleum ether (60–90°C, 500 ml × 3), chloroform (500 ml × 3) ethyl acetate (500 ml × 5) and n-BuOH (500 ml × 5). Reduced pressure for solvent recovery, then ethyl acetate extract (4.2 g) and n-BuOH fraction (35.0 g) were obtained.

The n-BuOH fraction (10 g) was dissolved in methanol, well-mixed with silica gel (20 g, 100–200 mesh), and used a dry packing method. The elution was carried out by a lower phase gradient of trichloromethane-methanol-water (15:1:0.2, 10:1:0.2, 8:1:0.2, 6:1:0.2, 3:1:0.2, 2:1:0.2) with 25 ml per time. Thin layer chromatography (TLC) with an alcoholic solution of sulfuric acid (5%) as chromogenic reagent was used. The same fractions were combined and concentrated by decompression to obtain fractions 2, 3, 4, and 5. Fraction 3 was dissolved in methanol, adding a proper amount of silica gel and applied to a column of silica. The column was washed with gradient elution of trichloromethane-methanol-water (6:1:0.2 → 2:1:0.2), combining the same constituents, and concentrating on decompression. Compound a (89 mg; compound-extract ratio: 0.0089%) could be obtained by eluting the Sephadex gel column with methanol-water (50:50). The same isolate method was applied in fraction four and got 38 mg of Compound b (compound-extract ratio: 0.0038%). Fraction 5 was separated by a column of silica with gradient elution of trichloromethane-methanol-water (4:1:0.2 → 2:1:0.2), combined the same constituents, and concentrated under decompression. Compound c (105 mg; compound-extract ratio: 0.0105%). could be obtained by eluting the Sephadex gel column with methanol-water (30:70).

The ethyl acetate extract (4.2 g) was dissolved in methanol, well-mixed with silica gel (5 g, 100–200 mesh), and used a dry packing method. The eluent gradient elution by ethyl acetate: petroleum ether (0–100%). TLC with an alcoholic solution of sulfuric acid (5%) as chromogenic reagent was used. The same constituents were combined and concentrated by decompression to obtain Compound d (50 mg; compound-extract ratio: 0.005%).

### The Methods for the Identification of Active Compounds From *Wosi*

#### Thin-Layer Chromatography

The acid hydrolysis compounds of *Wosi* and standard compound were performed on silica gel 60 A plates (Merck, Darmstadt, Germany). Samples were allowed to move in the mobile phase (hexane: AcOEt, 8:2) till 3/4th of the plate. Then the plates were dried in a current of air by mean of an air dryer. The coincidence of spots was observed under continuous UV spectrum and calculated the R_f_ value of the corresponding spots.

#### Mass Spectrometry Detection

MS spectra were recorded with a Waters Quattro Premier XE system using electrospray ionization (ESI) technique. MS was operated in negative ion mode with a collision energy of 5 V. The entrance and exit voltage were set at 2 and −10 V, respectively. The MS data were obtained in full scan mode (mass range 100–1,000 amu).

#### NMR Measurements

NMR experiment were performed at Bruker AVANCE 400 Fourier Transform spectrometer, operating at 400 MHz for ^1^H and 100 MHz for ^13^C. All the NMR measurement were made on 5 mm NMR tubes. For recording ^1^H NMR spectrum, solutions were prepared by dissolving 7 mg of the sample in 0.5 ml for MeOD-d4 while for ^13^C NMR spectrum about 10 mg of the *Wosi* compounds was dissolved in the same volume of the solvent. TMS was used as an internal standard. Chemical shifts (δ) are expressed in ppm relative to TMS. All solvents were evaporated below 40°C under reduced pressure.

### The Interaction of *Wosi* Compounds With COX-1 and COX-2 Enzymes via Molecular Docking.

The crystal structure of COX-1 enzyme with 2.6 Å resolution complex (PDB code: 3N8Y) and COX-2 enzyme with 1.73 Å resolution complex (PDB code: 3NT1) was obtained from the PDB database https://www.rcsb.org/. The 3D structure models of the main active compounds from *Wosi* were built and minimized by using the program ChemDraw 19.0, and then saved in mol2 format. As enzyme preparation, the ligands and water molecules were removed, then polar hydrogen atoms and Gasteiger charges were added by using AutoDockTools 1.56. Moreover, the docking input files of target proteins and ligands were prepared as PDBQT format by using the AutoDock Tools software. Finally, Molecular docking of compounds into the crystal structure of COX-1 enzyme (PDB code: 3N8Y) and COX-2 enzyme (PDB code: 3NT1) were performed via AutoDock Vina 1.12 software by Lamarckian genetic algorithm (Morris et al., 1998), it was used for docking against the receptors and to estimate the binding affinities (kcal mol^−1^). The Lamarckian genetic algorithm implemented in Auto Dock Vina was utilized as the key search protocol. There is a configuration file (config.txt) that the path to the receptors and ligands were set. The config.txt files contain the receptor file name, the ligand file name, the x, y, and z coordinate of the center of the grid box, the size of the grid box dimension and maximum number of binding poses to be generated for each dock. The parameters were the following: a grid box was prepared individually for COX-2 to cover the pocket with the main residues for enzyme binding site by maintaining the grid size at 82 × 70 × 62 Å in the x-, y-, and *z*-axis, respectively, and a grid size of x = 80 years = 70 z = 58 Å for the COX-1 enzyme, centered at coordinate x, y, z -33.802, -45.341, -24.413 (for COX-2) and 36.007, -52.132, -1.553 (for COX-1) respectively, with 1 Å grid spacing for all the enzymes. Auto Dock Vina was run using an exhaustiveness value of 120, and the final number of conformations generated was set as 20. The ligands were individually evaluated in silico against COX-2 enzyme and COX-1 enzyme in triplicates and the average of the best conformation was chosen with the lowest docked energy, based on complete docking search. Hydrogen bond and bond length were used as parameters to measure the interaction between COX-1 and ligand or COX-2 and ligand in PyMOL software.

### Validation of the Molecular Docking Method

In order to validate our docking-scoring procedure, the ligand Diclofenac was extracted from the crystal structure of COX-1 enzyme (PDB code: 3N8Y) and the ligand Naproxen was stripped from the crystal structure of COX-2 (PDB code: 3NT1). Then two ligands were docked back into the docking pocket of the relevant enzyme via the same conditions described above, respectively. The ligand structures of diclofenac and naproxen were stored in a PDBQT format as described above. We employed the same config.txt file for each complex in Auto Dock Vina docking to ensure the same parameters for comparison. Root-mean-square deviation (RMSD) between the best redocked conformation and the original conformation of the ligand was then calculated using the PyMOL software.

### *In Vitro* Cyclooxygenase Inhibition Assay

The inhibitory effects of three compounds from *Wosi* on COX activity was carried out measuring the synthesis of prostaglandin (PGE_2_) according to the manufacturer’s instructions provided with the commercial colorimetric COX inhibitor screening assay kit (Cayman test kit-560131; Cayman Chemical Company). The IC_50_ values (the concentration of the three compounds from *Wosi* causing 50% inhibition) were calculated from the concentration-inhibition response curves (duplicate determinations).

### Immunohistochemical Detection

The method of immunohistochemical staining for the ankle joint was as following the previous studies. Briefly, the sections were deparaffinized and dehydrated by xylene and ethanol. Endogenous peroxidase was quenched by H_2_O_2_ (3%). The 5% BSA was used for minimizing the non-specific adsorption for 30 min. The sections were incubated with primary antibodies, including anti-VCAM-1 and anti-COX-2 at overnight at 4°C, the slides were washed with TBS, and then incubated with polymerized HRP labeled anti-rabbit IgG for 30 min at 37°C. Finally, the sections were washed with TBS three times, and antibody binding was detected using 3,3′-diaminobenzidine tetrahydrochloride (DAB). Slides were counter-stained with hematoxylin solution. Stained sections were dehydrated and then each section was analyzed by microscope and image output system.

### Statistical Analysis

All results were expressed as the mean ± standard error of the mean (SEM). The one-way analysis of variance (ANOVA) was used to determine the level of significance followed by using GraphPad Prism 5.0 (GraphPad Software Inc., San Diego, CA, USA).

## Results

### Effects of *Wosi* Treatment on the Uric Acid Level in Hyperuricemia Rats

**FIGURE 1:**
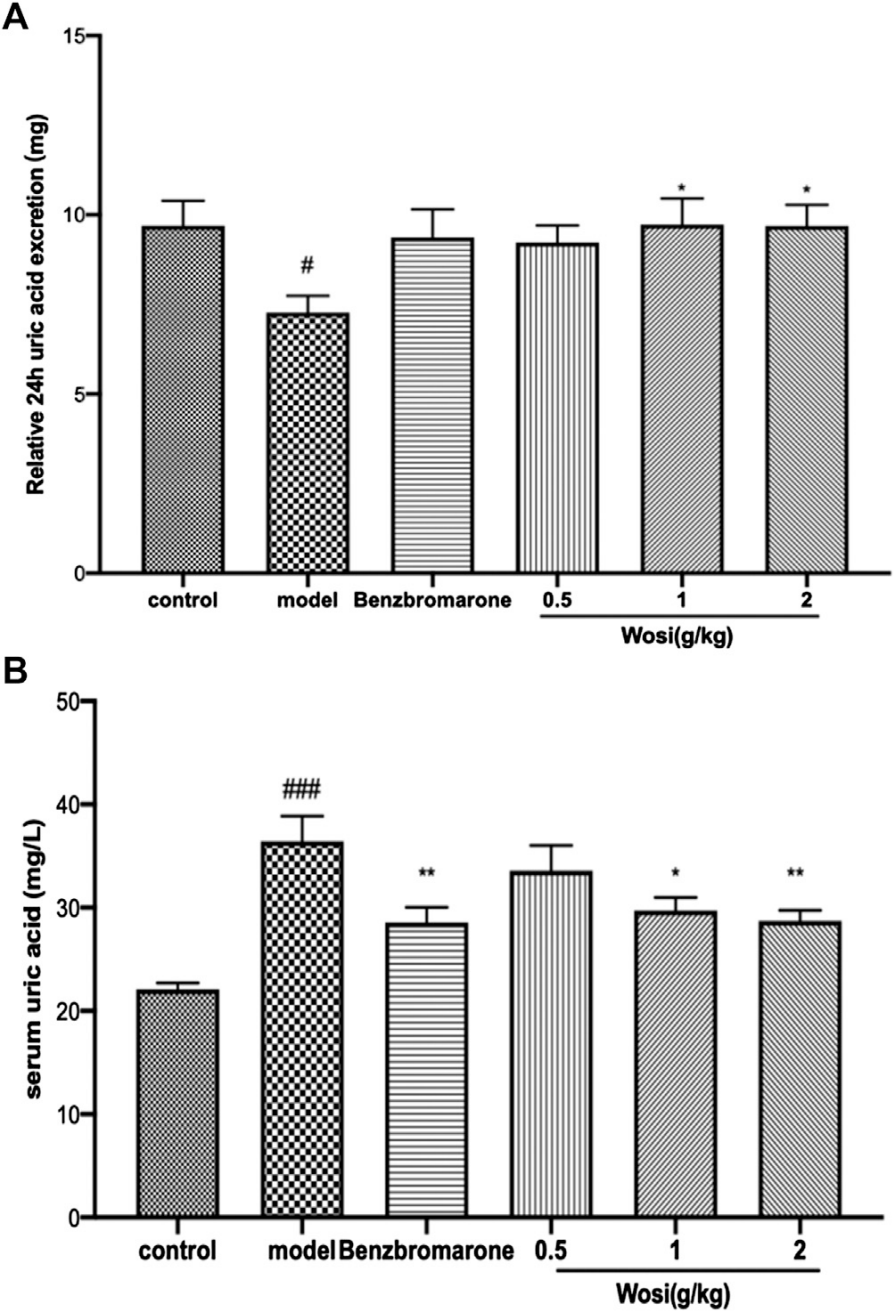
Impact of *Wosi* (0.5;1;2 g/kg) and benzbromarone (10 mg/kg) treatment on hyperuricemia rats. The 24-h urine excretion **(A)** and serum uric acid **(B)** were calculated. Data were presented as mean ± SEM for ten animals. #*p* < 0.01; ##*p* < 0.01 vs. control group; *p < 0.05, **p < 0.01 vs. model group.

The serum level of uric acid increased, and the urine uric acid excretion decreased markedly in the hyperuricemia rats when compared with the normal rats after 15 days, indicating that the model of hyperuricemia rat was successfully established. Following the therapeutic intervention, *Wosi* was demonstrated to reduce the serum levels of uric acid in a dose-dependent manner (Figure 1B) and increase the excretion of uric acid in the urine (Figure 1A). The serum level of uric acid and the urine uric acid excretion in the hyperuricemia rats treated with the positive control benzbromarone recovered to that of the normal animals. The effect of *Wosi* was found to be statistically significant after 5 days of treatment since the urine uric acid excretion and serum uric acid level of *Wosi*-treated groups were recovered to the level of normal animals or the Benzbromarone-treated group. A significant decrease was detected in the serum level of uric acid in the hyperuricemia rats treated with *Wosi* particularly at a high dose (2 g/kg/day). The above data showed that the effect of *Wosi* might serve as a therapeutic agent against hyperuricemia since the treatment was shown to reduce the serum level of uric acid and the urine uric acid excretion in the hyperuricemia experimental rat model and produce an effect comparable with the effect of Benzbromarone.

### Effect of *Wosi* on MSU-Induced Joint Swelling in Rats

**FIGURE 2:**
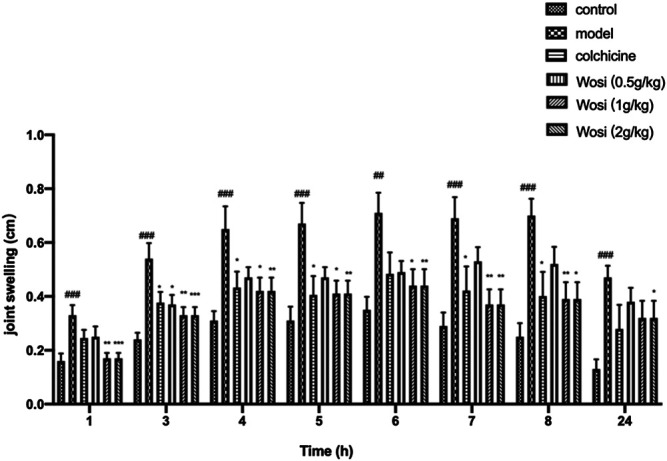
Effect of *Wosi* (0.5;1;2 g/kg) and colchicine (0.2 mg/kg) treatment on MSU crystal-induced joint swelling in rats at different time points. Values were shown as mean ± SEM for ten animals. ###p < 0.001 vs. control group; *p < 0.05; **p < 0.01; ***p < 0.001 vs. model group.

After the injection of MSU crystal, the joint swelling of the model rats continued increased compared with the control group, indicating that the acute gouty arthritis rat model was successfully established. Following therapeutic, *Wosi* was demonstrated to reduce the joint swelling in a dose-dependent manner (Figure 2). *Wosi*-treated group showed more effective than the positive control colchicine-treated group in joint swelling of MSU-induced rats. Especially in the first hour, the joint swelling of *Wosi*-treated groups was recovered the level of normal animals. The effect of *Wosi* was found to be statistically significant after 7 days treatment since the joint swelling of acute gouty arthritis rats treated with *Wosi* particularly at a high dose (2 g/kg/day) was decreased at all the time. The results were showed that the effect of *Wosi* might serve as a therapeutic agent against acute gouty arthritis, since the treatment was shown to attenuate the joint swelling in MSU-induced rats and produce an effect better than the effect of Colchicine.

### Effects of *Wosi* on the Inflammatory Cytokine Levels in MSU-Inducted Models.

**FIGURE 3:**
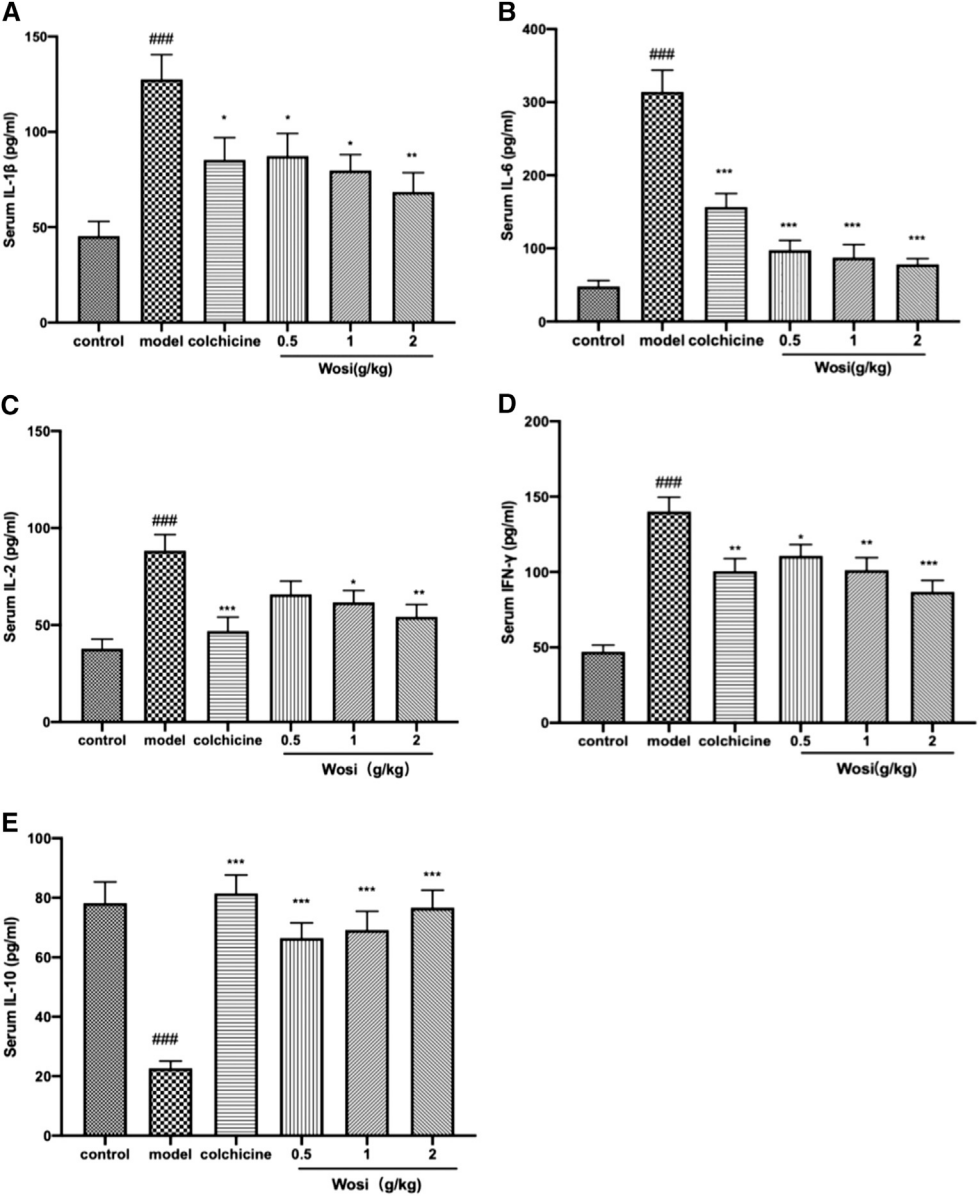
Effect of *Wosi* and colchicine treatment on inflammatory cytokines in the serum of MSU crystal-induced acute gouty arthritis rats. The serum level of inflammatory cytokines of experimental rats were quantified using ELISA kits as per manufacturer’s instructions. **(A)** IL-1β, **(B)** IL-6(B), **(C)** IL-2, **(D)** IFN-γ, and **(E)** IL-10. The values were expressed as mean ± SEM for ten animals. ###*p* < 0.001 vs. control group; *p < 0.05, **p < 0.01; ***p < 0.001 vs. model group.

The serum levels of pro-inflammatory cytokine (IL-2, IL-1β, IL-6, and IFN-γ) were significantly increased and anti-inflammatory cytokine IL-10 was found decreased markedly in MSU-induced rats when compared with normal control rats after 7 days (Figure 3). *Wosi* was demonstrated to reduce the serum levels of IL-1β, IL-2, and IFN-γ in a dose-dependent manner. The positive drug colchicine (0.2 mg/kg) also inhibited IL-2, IL-1β, IL-6, and IFN-γ secretion and increased IL-10 secretion in the serum. The effect of *Wosi* was found to be statistically significant after 7 days of treatment since the serum IL-6 level of *Wosi*-treated groups at all dosages was recovered to the level of normal animals. By contrast, treatment with *Wosi* suppressed the serum level of IL-6 in MSU-induced rats to greater extent than colchicine treatment when compared with MSU-induced rats alone. A significant increase was detected in the serum level of IL-10 in MSU-induced rats treated with *Wosi* at all dosages. In particular, the level of IL-10 recovered to the level of normal animals or the colchicine-treated group. The above data showed that *Wosi* might have potential ani-inflammatory property since the treatment was shown to suppress the serum levels of pro-inflammatory cytokine (IL-2, IL-1β, IL-6, and IFN-γ) and increase the level of IL-10.

### Structure Analysis of Active Ingredients of *Wosi*

Compound a was isolated as white crystals, and the Libermann-Burchard showed a positive reaction. After acid hydrolysis, the TLC identification showed that the aglycone was oleanolic acid. The [M-H]^−^ ion of compound a was detected at m/z 777.3056, indicating a molecular formula of C_42_H_66_O_13_ and the aglycone connected with two glycosyls. The fragment ions at 631.2922 [M-H-146]^−^, 455.2480 [M-H-146-176]^−^, indicating that the structure of rhamnose and glucuronic acid. In the spectrum of ^13^C-NMR, δ 123.69, 145.20 were the feature signal of olefinic carbons, δ181.90 was the carbonyl-c signal. In HNMR (MeOD-d4) spectrum, δ 1.15 (1H, s), 1.04 (1H, s), 0.94 (1H, s), 0.93 (1H, s), 0.90 (1H, s), 0.83 (1H, s), 0.80 (1H, s), δ 5.23 (1H, t) were fragment structure of oleanolic acid. The ^13^C-NMR spectrum showed two glucose terminal-group signals, δ 102.63, and 106.67 ppm, and identified the sugar conformation with rhamnose as α-L- and glucuronide as β-D-. The comparison of these data with literature led to the identification of compound a as a 3-O-[α-L-pyran rhamnose(1-3)-β-D-pyran glucuronic acid]-oleanolic acid (Supplementary Tables S2–S4).

Compound b was obtained as a white powdered crystalline. The [M-H]^−^ ion of compound was observed at m/z 793.36, indicating a molecular formula of C_42_H_66_O_14_ and the aglycone connected with two glycosyls. An additional O atom, compared to compound a, stated that it might have the structure of glucose and glucuronic acid. In the spectrum of ^13^C-NMR, δ 123.86, 144.85 were the feature signal of olefinic carbons, δ 178.07 was the carbonyl-c signal. In HNMR (MeOD-d4) spectrum, δ 1.17 (1H, s), 1.07 (1H, s), 0.97 (1H, s), 0.95 (1H, s), 0.93 (1H, s), 0.86 (1H, s), 0.81 (1H, s), δ 5.27 (1H, t) were fragment structure of oleanolic acid. The ^13^C-NMR spectrum showed two glucose terminal-group signals, and the signal of δ 91.09, 95.72, and 106.98 ppm identified the saccharic configuration and linking form. By comparing with the previous reports, compound b was identified as 3-O-(β-D-pyran glucuronic acid)-oleanolic acid-28-O-β-D-pyran glucoside (achyranthoside Ⅱ) (Supplementary Tables S2–S4).

The [M-H]^−^ ion of Compound c was observed at m/z 939.2986, indicating a molecular formula of C_48_H_76_O_18_ and the aglycone connected with three glycosyls, which stated that the structure of glucose, rhamnose, and glucuronic acid. In the spectrum of ^13^C-NMR, δ123.69, 145.20 were the feature signal of olefinic carbons, δ 181.90 was the carbonyl-c signal. In HNMR (MeOD-d4) spectrum, δ1.15 (1H, s), 1.04 (1H, s), 0.94 (1H, s), 0.93 (1H, s), 0.90 (1H, s), 0.83 (1H, s), 0.80 (1H, s), δ 5.23 (1H, t) were fragment structure of oleanolic acid. The ^13^C-NMR spectrum showed three glucose terminal-group signals, and the signal of δ 83.54, 90.90, 95.74, 102.63, and 106.67 ppm identified the saccharic configuration and linking form. Compound c was identified as 3-O-[α-L-pyran rhamnose(1-3)-β-D-pyran glucuronic acid]-oleanolic acid-28-O-β-D-pyran glucoside (achyranthoside I) by comparison of the physical and spectral data with the literature (Supplementary Tables S2–S4).

Compound d was isolated as white flaky crystals which were spotted on the same thin layer plate as the β-sitosterol reference substance. The R_f_ value and color development were the same in the three different solvent systems. The three solvent systems are petroleum ether: acetone (5:1); dichloromethane; n-hexane-ethyl: acetate (4:1), and the melting point did not decrease after mixing. The TLC identification showed that the compound d was *β*-sitosterol.

### Molecular Docking

**FIGURE 4:**
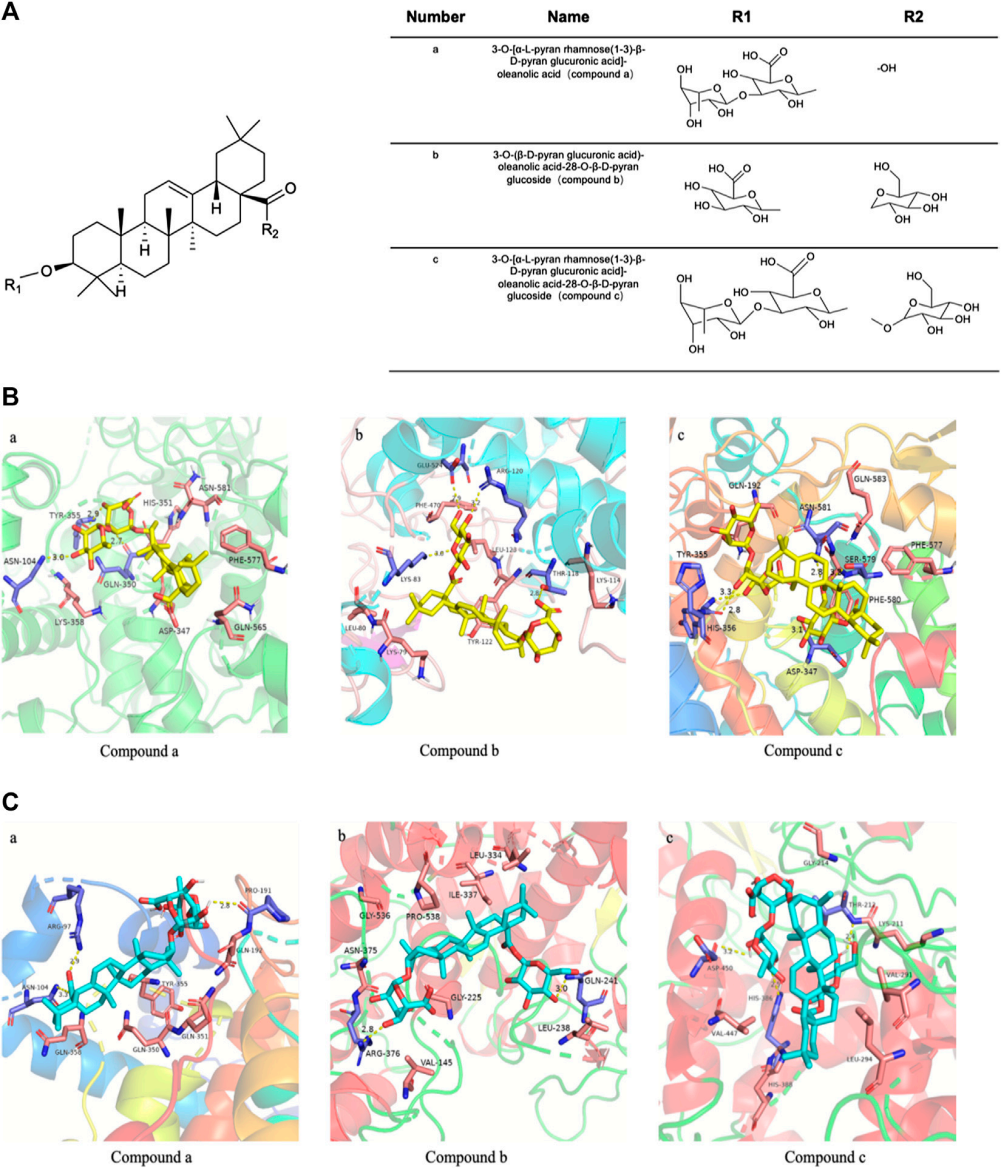
The chemical structure of the main biologically active compounds of *Wosi* **(A)**. Molecular docking **(B)** of *Wosi* main bioactive compounds (a, b, and c) with COX-2 enzyme (PDB code: 3NT1); Molecular docking **(C)** of *Wosi* main bioactive compounds (a, b, and c) with COX-1 enzyme (PDB code: 3N8Y).

To further beef up our understanding about the main compounds from *Wosi* against cyclooxygenase for anti-inflammatory properties, a series of compounds from *Wosi* which may have great potential were predicted by molecular docking. The hydrogen-bond interactions, bind affinity, bond length, active site residues, and orientation of the docked compounds within the active site were visualized (Figures 4B,C). Estimations of binding energy, H-bonds distance and interacting amino acids for each compound and target were shown in Tables 1. and 2. The negative and low values of affinity indicated a strong and favorable bonding between COX and the ligands in their conformations. The aminoacidic active site residues for cyclooxygenase are ARG-120, TYR-355 and GLU-524 residues, which involved in a hydrogen bond network. It will be contracted in the bottom area of the substrate binding site (Kozak et al., 2003; Marnett et al., 1999). The formation of interaction between these residues and the carboxylate of arachidonic acid (AA) is the main determinant of substrate binding (Malkowski et al., 2000). TRY-355, ASN-104, and GLN-350 residues in the binding pocket of COX-2 enzyme formed three hydrogen bonds with Compound a, with respective bond lengths of 2.9, 3.0, and 2.7 Å. It showed the possibility of hydrophobic interactions with residues of GLN-565, HIS-351, ASP-347, and PHE-577. These interactions could be checked on Figures 4B, a. The pentacyclic triterpene moiety of Compound b was trapped in the hydrophobic pocket, which was composed of TYR-122, LYS-79 and LEU-80. The formation of hydrogen bonds interaction between NSAIDs and ARG-120 is essential for inhibition (Blobaum et al., 2013). There were also hydrogen bonds between the saccharide moiety of Compound b and the key residues including GLU-524 (bonding length 2.9 Å), LYS-83 (bonding length 3.0 Å), THR-118 (bonding length 2.8 Å), and ARG-120 (bonding length 3.2 Å) for COX-2 (Figures 4B, b). In addition, Compound c also moderately interacted with other amino acid residues, through the hydrophobic interaction including PHE-580, PHE-577, GLN-583 and GLN-192, and hydrogen bonds including ASP-347, SER-579, ASN-581, HIS-356, and TYR-355 (hydrogen bonds at distance of 3.1, 3.2, 2.8, 2.8, and 3.3 Å, respectively) (Figures 4B, c). On the other hand, the COX-1 docking of Compound a showed three hydrogen bonds with PRO-191, ASN-104, ARG-97 at 2.8, 3.3, and 2.9 Å, respectively, while hydrophobic contacts have been observed from the surrounding residues including GLN-358, GLN-350, GLN-351, TYP-355 and GLN-192 (Figures 4C, a). Compound b showed two interaction with COX-1: one hydrogen bond with ARG-379 at distance of 2.8 Å, another hydrogen bond with GLN-241 at distance of 3.0 Å. It also showed the hydrophobic interactions with residues of ILE-337, PRO-538, GLY-225, and LEU-238 (Figures 4C, b). The binding pocket of COX-1 enzyme contain THR-212, HIS-386, ASP-450 formed hydrogen bonds with Compound c at distance of 2.4, 2.5, and 3.2 Å, respectively, through the hydrophobic interaction including HIS-388, VAL-291, and LYS-211 (Figures 4C, c). Amino acid active site residues for cyclooxygenase (ARG-120, TYR-355 and GLU-524) are approximately the same as the binding amino residues of cyclooxygenase inhibitors which reported in the literature (Luong et al., 1996; Malkowski et al., 2000; Kozak et al., 2003; Rowlinson et al., 2003). The saccharide moiety of the compounds may help improve their hydrophily, and the amino acids (ARG-120, TRY-355 and GLU-524) at the binding pocket, as well as the moderate interaction of hydrophobic and hydrogen bonds would therefore be essential for the stable conformation of the ligand–enzyme complexes.

**TABLE 1:** Interacting amino acids, H-bonds distance, and binding scores of COX-2 enzyme with *Wosi* Compound a, Compound b, and Compound c.

Name of the ligand	Binding affinity (kcal mol^−1^)	Number of hydrogen bonds	Distance (Å)	Residue
Compound a	−9.1	3	2.9	TYR-355
			3.0	ASN-104
			2.7	GLN-350
Compound b	−9.3	4	2.9	GLU-524
			3.0	LYS-83
			2.8	THR-118
			3.2	ARG-120
Compound c	−9.5	5	3.1	ASP-347
			3.2	SER-579
			2.8	ASN-581
			2.8	HIS-356
			3.3	TYR-355

**TABLE 2:** Interacting amino acids, H-bonds distance, and binding scores of COX-1 enzyme with *Wosi* Compound a, Compound b, and Compound c.

Name of the ligand	Binding affinity (kcal mol^−1^)	Number of hydrogen bonds	Distance (Å)	Residue
Compound a	−8.9	3	2.8	PRO-191
			3.3	ASN-104
			2.9	ARG-97
Compound b	−8.7	2	2.8	ARG-376
			3.0	GLN-241
Compound c	−8.5	3	2.4	THR-212
			2.5	HIS-386
			3.2	ASP-450

Our docking study showed that the binding affinity of the three main compounds for COX-2 was smaller than COX-1, this may indicate that the compounds show selective activity against COX-2 enzyme. The ligand-receptor fitting was best with the compound c with −9.5 kcal mol^−1^ for COX-2 and for COX-1 with −8.5 kcal mol^−1^, followed by compound b (COX-2 binding affinity −9.3 kcal mol^−1^ and COX-1 binding affinity −8.7 kcal mol^−1^), compound c showed the least binding affinity (COX-2 binding affinity −9.1 kcal mol^−1^ and COX-1 binding affinity −8.9 kcal mol^−1^). The above data disclosed that three compounds docked with favorable binding affinity to the COX-2 active cavity, when compared to dock with COX-1. In general, three compounds showed not only promising potential in inhibiting the COX-2, but also excellent selectivity over COX-1. It suggested that three active compounds from *Wosi* might work with less gastrointestinal side effect as COX-1 enzyme had ability to synthesis the gastroprotective prostaglandin synthesis in the stomach. The chemical structure of three efficient compounds from *Wosi* were showed in Figure 4A.

### Validation of the Molecular Docking Method

The method which has been widely used to confirm the reliability of the molecular docking is docking the co-crystallized ligand back to its corresponding receptor protein (Hevener et al., 2009). RMSD value was often used to measure the quality of reproductive binding pose by a computational method, such as molecular docking. RMSD value less than 2 Å was used to consider as a decent docking accuracy (Yusuf et al., 2008). For Diclofenac re-docking into the binding pocket of COX-1 (PDB code: 3N8Y) and the RMSD between the top pose and crystallographic pose is 0.712 Å. Meanwhile, Naproxen was dock back into the binding pocket of COX-2. The calculated RMSD for COX-2 is 0.180 Å. As shown in Supplementary Figures S1 and S2, Auto Dock Vina successfully dock COX-1inhibitor Diclofenac and COX-2 inhibitor Naproxen back into the binding sites of the relevant enzyme, respectively. The docked Diclofenac and Naproxen are almost completely superimposed on their corresponding crystal conformations. It shows that our molecular docking method is accurate, so we can perform virtual screening through Auto Dock Vina docking.

### *In Vitro* Cyclooxygenase Inhibition Assay

**FIGURE 5:**
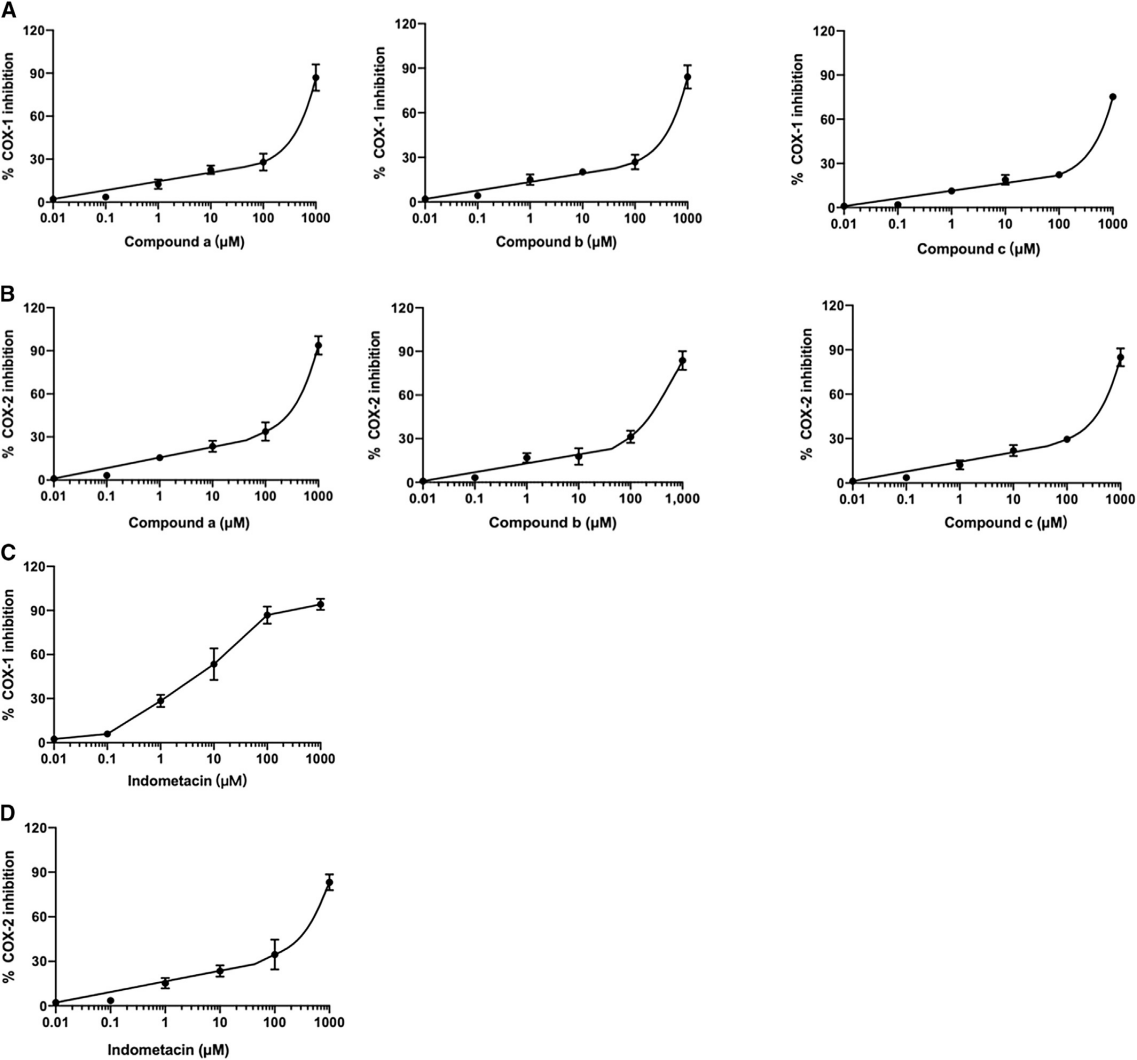
Effect of compounds (a, b, and c) from *Wosi* on COX-1 **(A)**, and COX-2 **(B)** and indomethacin on COX-1 **(C)**, and COX-2 (D) enzymes. The IC_50_ were determined using sigmoidal concentration-inhibition curves. The concentration used for the calculation of IC50 values ranged from 0.01 to 550 μM.

The ability of three compounds from *Wos*i against COX-1/2 enzymes was evaluated using colorimetric COX inhibitor screening assay and the results obtained were listed in Table 3. Three compounds (a, b, c) from *Wosi* showed a significant COX-2 inhibitory effect, directly on the enzyme activity, with IC_50_ value of 297.8, 294.2, 384.5 μM, respectively. Meanwhile, the COX-2/COX-1 selectivity ratio of 0.67, 0.60, 0.65, respectively. The result is in accordance with the molecular docking. The standard reference drug indomethacin inhibited COX-1 and COX-2 enzymes with IC_50_ values of 6.8 and 232.8 μM, respectively with the COX-2/COX-1 selectivity ratio of 34 (Table 3; Figure 5). These data are consistent with the literature reports (Ringbom et al., 1998).

**TABLE 3:** IC_50_ values (μM) and calculated selectivity ratio (COX-2/COX-1 ratio) of compounds (a, b, c) from *Wosi*.

Compound	IC_50_ (μM)		COX-2/COX-1
	COX-2	COX-1	
Compound a	297.8	442.6	0.67
Compound b	294.2	488.1	0.60
Compound c	348.5	537.9	0.64
Indomethacin	232.8	6.8	34

### Effects of *Wosi* on COX-2 and VCAM-1 Protein Expression of Synovial Tissue in the Ankle Joint

**FIGURE 6:**
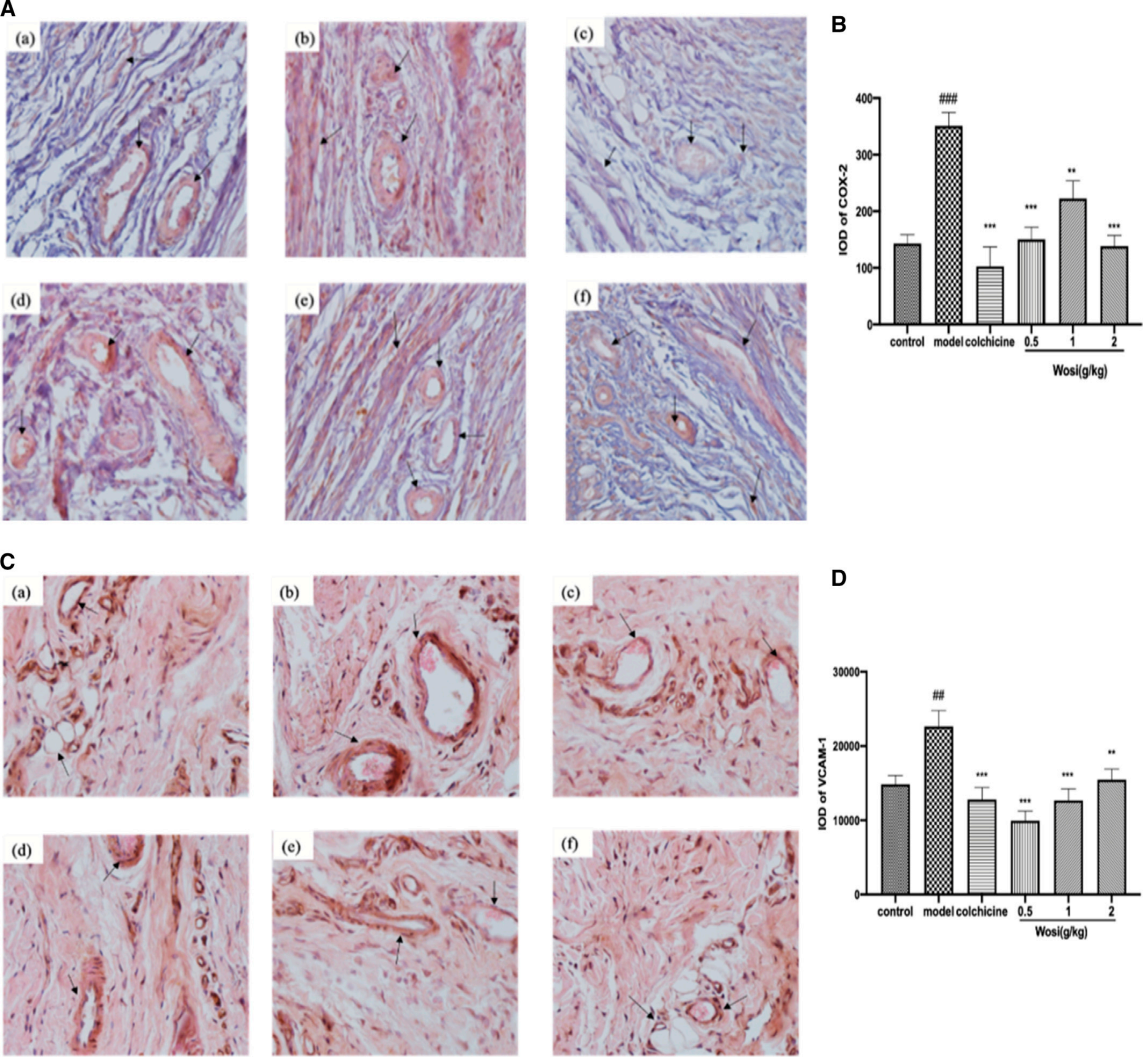
Effect of *Wosi* and colchicine treatment on the level of **(A)** COX-2 and **(C)** VCAM-1 proteins were detected by immunohistochemistry (IHC). Representative photomicrographs of staining (×20) demonstrating the synovial tissue sections from ankle joints of experiments rats. Treatment of groups are as follow: (a) control group; (b) model group; (c) colchicine group (0.2 mg/kg); (d) *Wosi* group (0.5 g/kg); (e) *Wosi* group (1 g/kg); (f) *Wosi* group (2 g/kg). The integrated optical density (IOD) of COX-2 (B) and VCAM-1 (D) IHC-stained materials were analyzed utilizing the Image pro plus 7.0 software. IOD = optical intensity of positive cells × area of positive cells. The value was expressed as mean ± SEM for ten animals. ###p < 0.001 vs. control group; **p < 0.01; ***p < 0.001 vs. model group.

To confirm the potential anti-inflammatory properties of *Wosi*, the protein expression of COX-2 and VCAM-1 in synovial tissue sections from ankle joints of MSU-induced rats were detected via immunohistochemistry analysis (Figures 6A,C). Then the COX-2 and VCAM-1 results were quantified via using Image pro plus 7.0 software which showing in Figures 6B,D. After the injection of MSU crystal, the protein expression of COX-2 and VCAM-1 continue to increase in MSU-induced rats when compared with normal control group (*p* < 0.001, *p* < 0.01, respectively). The protein expression of COX-2 and VCAM-1 in MSU-induced rats treated with the positive control colchicine recovered to that of the normal animals. A significant decrease was detected in the protein expression of COX-2 in MSU-induced rats treated with *Wosi* particularly at dose (0.5 and 2 g/kg/day). The effect of *Wosi* was found to be statistically significant after 7 days of treatment since the VCAM-1 protein expression of *Wosi*-treated groups was recovered to the expression of normal animals. The above data implied that *Wosi* had the potential ability to cure the acute gouty arthritis, which acted by influencing the COX-2 and VCAM-1 signaling.

## Discussion

Due to an unhealthy diet, the incidence of gouty arthritis and hyperuricemia is increasing year by year worldwide. Hyperuricemia is a disease in which purine metabolism is disordered due to increased serum uric acid or insufficient uric acid by high-purine diet (Kaminska-Pajak et al., 2016). When uric acid in the blood gradually accumulates to a concentration higher than 7.0 mg/dl, it causes sodium urate crystals deposition in the joints and other tissues which can lead to acute gouty arthritis (Pillinger et al., 2007). The deposition of MSU crystals in the joints appears to activate innate host defense mechanisms and triggers robust inflammation to release the proinflammatory cytokines and further promotes inflammation (Landis and Haskard, 2001; Schauer et al., 2014). Anti-inflammatory and anti-hyperuricemia properties are important for compounds intended for acute gouty arthritis, but none of the clinically available medicines has both effects at the same time. In this study, it identified three oleanolic acid glycosides from Yi nationality herbal formula *Wosi* and performed the anti-inflammatory and anti-hyperuricemia properties of *Wosi* based on hyperuricemia rats and acute gout arthritis model.

In the rats with hyperuricemia, the concentration of serum uric acid directly reflects the severity of hyperuricemia. In this study, it demonstrated the potent uricosuric effect of *Wosi* by increasing uric acid excretion for 24 h, thus decreasing the concentration of uric acid in the serum. In short, the results suggested that *Wosi* might serve as a therapeutic agent against hyperuricemia since the treatment was shown to reduce the serum level of uric acid and the urine uric acid excretion in the hyperuricemia experimental rat model.

The edema observed on animals that received vehicle after MSU crystal injection was considered as the inflammation (Busso and So, 2010; Manger et al., 2012; Cronstein and Sunkureddi, 2013). It was used as a reference and compared to other treatments to evaluate the anti-inflammatory activity*. Wosi* treatment successfully suppressed joint swelling in the ankles of rats stimulated by monosodium urate crystals, indicating its activity against gouty arthritis. In the rats with acute gouty gout arthritis, it triggered an inflammation reaction in macrophage and T lymphocyte to release inflammatory factors after monosodium urate crystals were injected (Kapoor et al., 2011), which helped to amplify the inflammation (Hugle and Krenn, 2016). The balance between pro-inflammatory and anti-inflammatory cytokines determined the degree and extent of inflammation. IFN-γ producing Th1 cells appear to be important in the inflammatory phase of acute gouty arthritis (Singh et al., 2007). IL-2 is a pro-inflammatory cytokine referred to as T cell growth factor that plays a central role in the therapeutic manipulation of the immune system (Yudoh et al., 2000). MSU-induced rats treated with *Wosi* (0.5, 1, and 2 g/kg) demonstrated a substantial decrease in the expression of pro-inflammatory (Th1) cytokines (i.e., IFN-γ and IL-2). In addition to enhanced expression of IL-10, which is anti-inflammatory Th2 cytokines. It showed that *Wosi* treatment might modulate the properties of T cells. The production of IL-10 is found to inhibit the release of pro-inflammatory cytokines such as IL-1β, and IL-6 (Waseem et al., 2008). IL-1β is one of the most widely studied members of the IL-1 family which can promote the migration of activated macrophage and neutrophils in the joints of MSU-induced rats (Oliveira et al., 2008). Moreover, IL-6 is a very important cytokine in inflammation, especially active in acute gouty arthritis, which can participate in the early activation of T-cell. The result also indicated that *Wosi* significantly reduced the levels of pro-inflammatory cytokines including IL-6 and IL-1β in MSU-induced rats. The present findings demonstrated that *Wosi* possessed anti-inflammatory effect by inhibiting the secretion of pro-inflammatory cytokines and increasing the release of the anti-inflammatory cytokines, it might be regarded as useful tool for the treatment of acute gouty arthritis. We screened a series of compounds from *Wosi* against cyclooxygenase for anti-inflammatory properties using molecular docking, among which three compounds (3-O-[*α*-L-pyran rhamnose(1-3)-*β*-D-pyran glucuronic acid]-oleanolic acid, 3-O-(*β*-D-pyran glucuronic acid)-oleanolic acid-28-O-β-D-pyran glucoside, and 3-O-[*α*-L-pyran rhamnose(1-3)-*β*-D-pyran glucuronic acid]-oleanolic acid-28-O-*β*-D-pyran glucoside) showed not only promising potential in inhibiting the COX-2, but also excellent selectivity over COX-1. Three compounds from *Wosi* are three oleanolic acid glycoside derivatives. The triterpenoids ursolic acid and oleanolic acid are known to possess anti-inflammatory activities *in vitro* and *in vivo* (Liu, 1995; Checker et al., 2012; Lee et al., 2013). There is literature reported that ursolic acid and oleanolic acid are the selective inhibitors of cyclooxygenase-2 catalyzed prostaglandin biosynthesis (Ringbom et al., 1998). As a proinflammatory enzyme, COX-2 plays an important effect on acute gouty arthritis, which is associated with the generation of PGE_2_ (Nuki, 2008). To confirm the potential anti-inflammatory properties of *Wosi* which is correlated with the *in-silico* data. Firstly, three compounds from *Wosi* were found to possess preferential selectivity toward inhibiting COX-2 enzyme over COX-1 which based on *in-vitro* enzyme inhibition assays. These data are consistent with the literature (Ringbom et al., 1998). Then COX-2 could be significantly downregulated after *Wosi* treatment in synovial tissue sections from ankle joints of experiments rats which has been demonstrated by immunohistochemical assay. It is showed that Yi nationality herbal formula *Wosi* could inhibit COX-2 with preferential selectivity to achieve the potential anti-inflammatory properties. VCAM-1 is an important adhesion factor and belongs to the immunoglobulin family of adhesion factors. Some research found that VCAM-1 plays an important role in adhesion, infiltration, and migration across the endothelium (Cook-Mills et al., 2011). It is mainly involved in the infiltration of monocytes and lymphocytes into the inflammatory site (Chapman et al., 1997). In human vascular smooth muscle cells, COX-2 can regulate VCAM-1 and ICAM-1 expression to limit inflammatory response (Bishop-Bailey et al., 1998). In this study, it also confirmed that *Wosi* treatment could reduce the expression of VCAM-1 protein in synovial tissue sections from ankle joints by immunohistochemical assay. In short, combined with the above molecular docking results, three oleanolic acid glycosides were implied as mainly efficient compounds in Yi nationality herbal formula *Wosi* for arthritis therapy via selectively influencing COX-2 and VCAM-1 signaling.

## Conclusion

In summary, the current study provided evidence that the main active compound from *Wosi* showed potential anti-hyperuricemia and anti-inflammatory activities. Firstly, *Wosi* treatment showed the main anti-hyperuricemia effect by directly reduced the uric acid in serum and increased uric acid excretion in the urine. Secondly, *Wosi* treatment significantly decreased joint swelling in MSU-induced rats. The inhibition of IL-2, IL-1β, IL-6, and IFN-γ secretion and IL-10 increased in the serum were observed. Furthermore, the study also focuses on the screening of the main compounds from *Wosi* against cyclooxygenase for anti-inflammatory properties using molecular docking. Three compounds from *Wosi* (3-O-[α-L-pyran rhamnose(1-3)-β-D-pyran glucuronic acid]- oleanolic acid, 3-O-(β-D-pyran glucuronic acid)-oleanolic acid-28-O-β-D-pyran glucoside, and 3-O-[α-L-pyran rhamnose(1-3)-β-D-pyran glucuronic acid]-oleanolic acid-28-O-β-D-pyran glucoside) showed not only promising potential in inhibiting the COX-2, but also excellent selectivity over COX-1. Meanwhile, the cyclooxygenase inhibition assay *in-vitro* confirmed that three compounds from *Wosi* were found to possess preferential selectivity toward inhibiting COX-2 enzyme over COX-1. The downregulated protein expression of COX-2 and VCAM-1 were also confirmed *in-vivo* by immunohistochemistry analysis after the *Wosi* treatment. In short, three oleanolic acid glycosides were implied as mainly efficient compounds in Yi nationality herbal formula *Wosi* for arthritis therapy via selectively influencing COX-2 and VCAM-1 signaling. The obtained evidence may help to guide the scientific treatment and in-depth exploration of Yi nationality herbal formula *Wosi* for acute gouty arthritis and related disease.

This study has limitation that will be addressed in the ongoing experiments. Yi nationality herbal formula *Wosi* contained high concentration of the oleanolic acid glycoside derivatives, although *Wosi* displayed better anti-inflammatory and anti-hyperuricemia activities, it failed to clarify which oleanolic acid glycoside derivatives of *Wosi* were effective against acute gout arthritis based on present data. It might be related to the synergistic effects of multi-effective components.

## Data Availability Statement

The raw data supporting the conclusions of this article will be made available by the authors, without undue reservation.

## Author Contributions

YL and PL designed the study and obtained financial support. J-ZZ and X-YC performed the experiments. X-YC wrote the manuscript. L-ML and LH carried out the data analysis. Q-ZY and Y-JW critically reviewed the manuscript. All the authors read, discussed and approved the final manuscript.

## Conflict of Interest

The authors declare that the research was conducted in the absence of any commercial or financial relationships that could be construed as a potential conflict of interest.
