# Supplementary material for: Identification of Active Compounds From Yi Nationality Herbal Formula Wosi Influencing COX-2 and VCAM-1 Signaling
Source: Front Pharmacol. 2020 Nov 9;11:568585. doi: 10.3389/fphar.2020.568585 (PMC7797783; doi:10.3389/fphar.2020.568585)

Supplementary Figure 1. Superposition of the structures of the best redocked conformation Diclofenac (magenta) and the original conformation of the ligand (green) within the active site pocket of COX1 (PDB code: 3N8Y). The calculated RMSD is 0.712 Å.

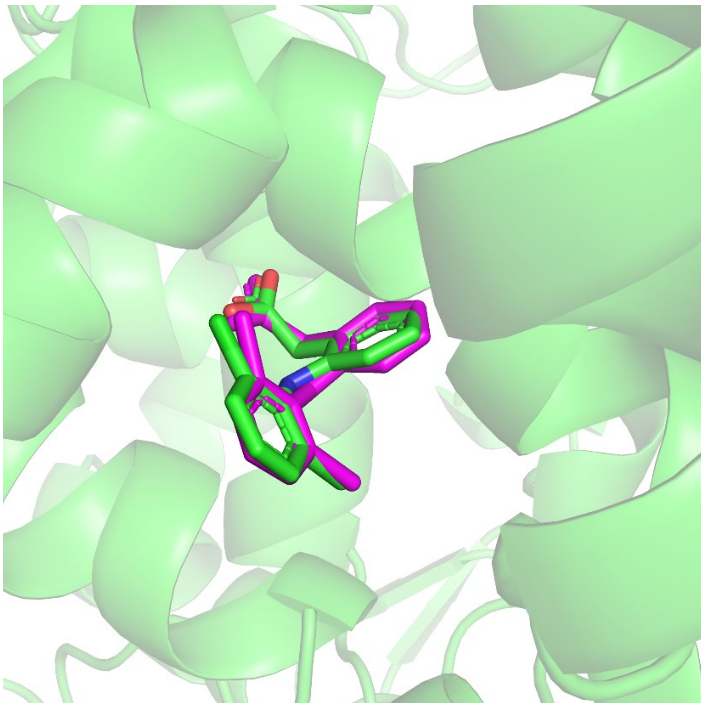

Supplementary Figure 2. Superposition of the structures of the best redocked conformation Naproxen (magenta) and the original conformation of the ligand (green) within the active site pocket of COX2 (PDB code: 3NT1). The calculated RMSD is 0.180 Å.

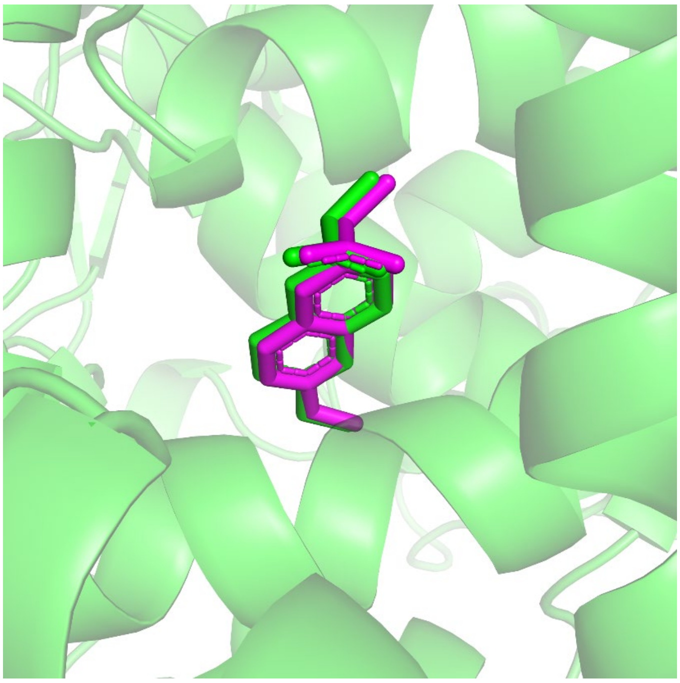

Supplement: Supplementary file 1 [file Image1.pdf]
