# Supplementary material for: Identification of Active Compounds From Yi Nationality Herbal Formula Wosi Influencing COX-2 and VCAM-1 Signaling
Source: Front Pharmacol. 2020 Nov 9;11:568585. doi: 10.3389/fphar.2020.568585 (PMC7797783; doi:10.3389/fphar.2020.568585)
Supplement: Supplementary file 2 [file Table1.docx]

**Table 1** The botanical compositions in *Wosi* preparation

| **No.** | **Collecting spots** | **Origins** | **Specimen No.** |
| --- | --- | --- | --- |
| S1 | Xicang, Sichuan | *Cyathula capitata* (Wall.) Moq. | 2013100401 |
| S2 | Xicang, Sichuan | *Sargentodoxa cuneata* (Oliv.) Rehd.et Wils. | 2013100402 |
| S3 | Xicang, Sichuan | *Dipsacus asperoides* C. Y. Cheng et T. | 2013100403 |
| S4 | Xicang, Sichuan | *GaultherialeucocarpaBl*.var.*crenulata* T. Z. Hsu | 2013100404 |
| S5 | Xicang, Sichuan | *Dioscorea collettii* Hook. f. var. *hypoglauca (Palibin)* C. T. | 2013100405 |

**T****able 2** ^13^C-NMR spectroscopic data for sapogenin（100MHz, MeOD-d4）

| **C** | **Compound a** | **Compound b** | **Compound c** | **C** | **Compound a** | **Compound b** | **Compound c** |
| --- | --- | --- | --- | --- | --- | --- | --- |
| 1 | 39.79 | 39.82 | 39.84 | 16 | 24.02 | 24.00 | 24.00 |
| 2 | 28.59 | 28.52 | 28.57 | 17 | 47.27 | 47.26 | 47.24 |
| 3 | 91.04 | 91.09 | 90.90 | 18 | 40.60 | 40.73 | 40.74 |
| 4 | 40.60 | 40.73 | 40.74 | 19 | 47.27 | 47.26 | 47.24 |
| 5 | 57.02 | 57.03 | 57.03 | 20 | 31.65 | 31.57 | 31.57 |
| 6 | 17.92 | 17.75 | 17.89 | 21 | 34.93 | 34.92 | 34.94 |
| 7 | 33.62 | 33.54 | 33.53 | 22 | 33.85 | 33.97 | 33.99 |
| 8 | 40.19 | 40.20 | 39.84 | 23 | 28.87 | 28.92 | 28.93 |
| 9 | 48.61 | 48.03 | 48.05 | 24 | 17.07 | 17.00 | 17.05 |
| 10 | 37.92 | 37.91 | 37.91 | 25 | 16.00 | 16.06 | 16.07 |
| 11 | 24.02 | 24.00 | 24.00 | 26 | 17.77 | 17.75 | 17.77 |
| 12 | 123.69 | 123.86 | 123.88 | 27 | 26.47 | 26.35 | 26.34 |
| 13 | 145.20 | 144.85 | 144.83 | 28 | 181.90 | 178.07 | 178.10 |
| 14 | 42.76 | 42.62 | 42.63 | 29 | 33.85 | 33.97 | 33.99 |
| 15 | 28.59 | 28.92 | 28.93 | 30 | 24.57 | 24.59 | 24.59 |

**Table 3** ^13^C-NMR spectroscopic data for monose in Saponine（100MHz, MeOD-d4）

| **C** | **28- O -Glc** | | | **3-O-GlcA** | | | **2′*-α*-L-Rham** | | |
| --- | --- | --- | --- | --- | --- | --- | --- | --- | --- |
|  | **Compound a** | **Compound b** | **Compound c** | **Compound a** | **Compound b** | **Compound c** | **Compound a** | **Compound b** | **Compound c** |
| 1 |  | 95.72 | 95.74 | 106.67 | 106.98 | 106.67 | 102.63 |  | 102.54 |
| 2 |  | 73.93 | 74.16 | 74.10 | 73.93 | 73.93 | 69.92 |  | 71.14 |
| 3 |  | 78.31 | 78.32 | 83.67 | 77.73 | 83.54 | 72.28 |  | 72.31 |
| 4 |  | 71.11 | 71.14 | 72.34 | 73.32 | 73.32 | 72.39 |  | 72.43 |
| 5 |  | 78.72 | 78.71 | 76.09 | 75.34 | 76.17 | 69.92 |  | 69.86 |
| 6 |  | 62.41 | 62.45 | 181.2 | * | 175.02 | 17.92 |  | 17.89 |

**Table 4** ^1^H-NMR spectroscopic data for sapogenin and monose in Saponine（400MHz, MeODd4）

| **H** | **Sapogenin** | | | | | |
| --- | --- | --- | --- | --- | --- | --- |
|  | **Compound a** | | **Compound b** | | **Compound c** | |
| 12 | 5.23 | 1 H（s） | 5.27 | 1 H（s） | 5.25 | 1 H（s） |
| 23 | 1.04 | 3 H（s） | 1.07 | 3 H（s） | 1.04 | 3 H（s） |
| 24 | 0.80 | 3 H（s） | 0.81 | 3 H（s） | 0.80 | 3 H（s） |
| 25 | 0.94 | 3 H（s） | 0.97 | 3 H（s） | 0.95 | 3 H（s） |
| 26 | 0.83 | 3 H（s） | 0.86 | 3 H（s） | 0.84 | 3 H（s） |
| 27 | 1.15 | 3 H（s） | 1.17 | 3 H（s） | 1.16 | 3 H（s） |
| 29 | 0.93 | 3 H（s） | 0.95 | 3 H（s） | 0.93 | 3 H（s） |
| 30 | 0.90 | 3 H（s） | 0.93 | 3 H（s） | 0.91 | 3 H（s） |
|  | 3-O-GlcA1-H | | | | | |
|  | 4.35 | 1H(s) | 4.39 | 1H(d，J=8.0 Hz) | 4.35 | 1H(d，J=8.0 Hz) |
|  | 2′*-α*-L-Rham1-H | | | | | |
|  | 5.16 | 1H(s) |  |  | 5.18 | 1H(s) |
|  | 28- O –Glc1-H | | | | | |
|  |  |  | 5.40 | 1H(d，J=8.0 Hz) | 5.38 | 1H(d，J=8.0 Hz) |

**Table 5** IC50 values (mg/L) and calculated selectivity ratio (COX-2/COX-1 ratio) of two fractions of 80% ethanol extract and n-BuOH fraction from *Wosi*

| **Groups** | **IC_50_ (mg/L)** | | COX-2/COX-1 |
| --- | --- | --- | --- |
|  | COX-2 | COX-1 |  |
| 80% ethanol extract | 202.0 | 223.1 | 0.91 |
| n-BuOH fraction | 301.5 | 391.3 | 0.77 |
